# Kidney tubule iron loading in experimental focal segmental glomerulosclerosis

**DOI:** 10.1038/s41598-022-05261-4

**Published:** 2022-01-24

**Authors:** Rachel P. L. van Swelm, Sanne Beurskens, Henry Dijkman, Erwin T. G. Wiegerinck, Rian Roelofs, Frank Thévenod, Johan van der Vlag, Jack F. M. Wetzels, Dorine W. Swinkels, Bart Smeets

**Affiliations:** 1 Translational Metabolic Laboratory (TML 830), Department of Laboratory Medicine, Radboud University Medical Center, Radboud Institute for Molecular Life Sciences, P.O. Box 9101, 6500 HB Nijmegen, The Netherlands; 2 Department of Pathology, Radboud University Medical Center, Radboud Institute for Molecular Life Sciences, Nijmegen, The Netherlands; 3 Institute of Physiology, Pathophysiology & Toxicology, Center for Biomedical Training and Research (ZBAF), University of Witten/Herdecke, Witten, Germany; 4 Department of Nephrology, Radboud University Medical Center, Radboud Institute for Molecular Life Sciences, Nijmegen, The Netherlands; 5 Department of Nephrology, Radboud University Medical Center, Radboud Institute for Health Sciences, Nijmegen, The Netherlands

**Keywords:** Kidney diseases, Nephrology, Chronic kidney disease

## Abstract

Kidney iron deposition may play a role in the progression of tubulointerstitial injury during chronic kidney disease. Here, we studied the molecular mechanisms of kidney iron loading in experimental focal segmental glomerulosclerosis (FSGS) and investigated the effect of iron-reducing interventions on disease progression. Thy-1.1 mice were injected with anti-Thy-1.1 monoclonal antibody (mAb) to induce proteinuria. Urine, blood and tissue were collected at day (D)1, D5, D8, D15 and D22 after mAb injection. Thy-1.1 mice were subjected to captopril (CA), iron-deficient (ID) diet or iron chelation (deferoxamine; DFO). MAb injection resulted in significant albuminuria at all time points (p < 0.01). Kidney iron loading, predominantly in distal tubules, increased in time, along with urinary kidney injury molecule-1 and 24p3 concentration, as well as kidney mRNA expression of *Interleukin-6* (*Il-6)* and *Heme oxygenase-1* (*Ho-1)*. Treatment with CA, ID diet or DFO significantly reduced kidney iron deposition at D8 and D22 (p < 0.001) and fibrosis at D22 (p < 0.05), but not kidney *Il-6*. ID treatment increased kidney *Ho-1* (p < 0.001). In conclusion, kidney iron accumulation coincides with progression of tubulointerstitial injury in this model of FSGS. Reduction of iron loading halts disease progression. However, targeted approaches to prevent excessive kidney iron loading are warranted to maintain the delicate systemic and cellular iron balance.

## Introduction

Chronic kidney disease (CKD) has a global estimated prevalence between 11 and 13%^1^. The major risk factor of disease progression is proteinuria, which is strongly associated with tubulointerstitial (TI) injury; the best histological hallmark of disease progression^2,3^. Treatment of proteinuric nephropathies is primarily aimed at reducing proteinuria by controlling blood pressure using, preferentially, angiotensin-converting enzyme (ACE) inhibitors^4,5^. However, reduction in proteinuria by such treatments is not adequate in many patients, leading to persistent proteinuria and progressive CKD^6^. There is an unmet need for effective treatment modalities for patients with persistent proteinuria, which could be addressed by interventions focused on preventing TI injury.

One of the mechanisms proposed to be involved is the accumulation of iron^7^. Increased concentrations of urinary and kidney iron were observed in various animal models of CKD^8–15^. In clinical studies, increased urinary iron concentrations were reported in patients with membranous glomerulonephritis, focal segmental glomerulosclerosis (FSGS) and diabetic nephropathy^16–19^. Kidney iron deposition was demonstrated in biopsies of patients with various types of proteinuric CKD^20–23^. Taken together, the animal and clinical studies suggest a role for iron in the onset and progression of CKD. However, the molecular mechanisms of kidney iron deposition and injury remain to be elucidated. Increased amounts of transferrin-bound iron (TBI) reach the tubular lumen in proteinuria, which can be reabsorbed via endocytosis in the proximal tubules (PT) by megalin or the transferrin receptor 1 (TfR1), or in the distal tubules (DT) by the 24p3 receptor (24p3R/NGALR/Lcn2-R) or TfR1^7,24,25^. Iron that is reabsorbed by tubular epithelial cells is stored intracellularly by ferritin, transported to iron-consuming organelles, such as the mitochondria, via chaperones, or exported to the blood through the only known mammalian iron exporter ferroportin (FPN). Excess iron, however, causes oxidative stress through the production of hydroxyl radicals via the Fenton and Haber/Weiss catalytic reactions. The formation of reactive oxygen species may, in turn, lead to inflammatory responses, ultimately leading to fibrosis^9,10,26^.

Previous studies reported beneficial effects of reducing kidney iron exposure on kidney injury; application of iron-deficient diets or iron chelation therapy reduced kidney iron loading, kidney injury and/or proteinuria in various murine models of CKD^8–11,13,15,27–29^. Only one clinical study to date reported a beneficial effect of iron chelation by deferiprone in patients with biopsy-proven glomerulopathy and diabetic nephropathy, leading to reduced proteinuria^30^. From this study it could not be concluded whether chelation prevented disease progression. Despite the promising results obtained by the studies summarized above, it is still unclear if reduction in iron exposure is sufficient to halt disease progression in persistent proteinuria. Moreover, a targeted approach to reduce kidney tubular iron loading might be necessary for chronic treatment of CKD patients, since long-term chelation therapy is associated with nephrotoxicity^31^.

Here, we aimed to investigate the mechanisms of tubular iron loading in an experimental model of FSGS, the Thy-1.1 mouse model. These mice ectopically express Thy-1.1 protein on their podocytes and develop persistent proteinuria after injection of a monoclonal antibody (mAb) directed against the Thy-1.1 transgene^32^. Severe glomerular sclerotic lesions and TI fibrosis develop within 21 days after mAb injection, which allows assessment of disease progression within a relatively short time frame^33^. In this model, we also investigated the effect of iron reducing interventions on kidney iron deposition and progression of kidney injury.

## Results

### Distal tubular iron accumulation in experimental FSGS

**Figure 1:**
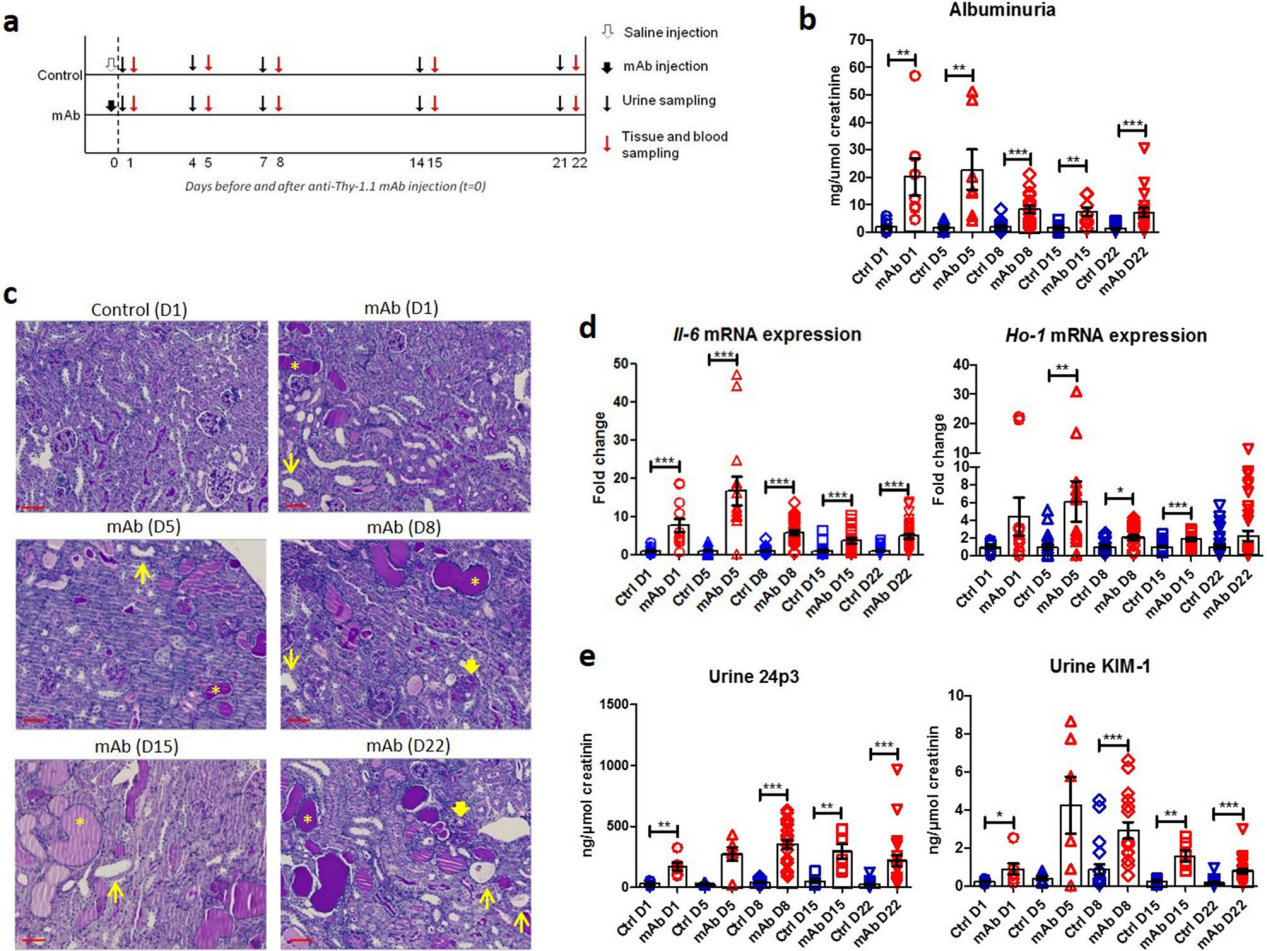
Progressive kidney injury in Thy-1.1 mice after mAb injection. Schematic overview of the experimental setup (**a**); transgenic Thy-1.1 mice were injected with anti-Thy-1.1 antibody (mAb) or saline as control (Ctrl) and sacrificed at the indicated time points after 24 h urine collection. Injection of mAb resulted in significantly increased albuminuria compared to control at all time points (**b**). Representative images of kidney PAS staining show progressive kidney injury in mAb-injected mice characterized by tubule dilatation (narrow arrows) and loss of brush border, proteinuric cast formation (asterisks) and glomerular sclerosis (thick arrows) (**c**). Kidney mRNA expression level of *Il-6* and *Ho-1* (**d**) and urine excretion of kidney injury markers 24p3 and KIM-1 (**e**), demonstrate significant kidney injury in mAb-injected mice compared to control. *p < 0.05, **p < 0.01, ***p < 0.001 compared to control using Mann–Whitney t-test; *D* day, *Ctrl* control, *mAb* monoclonal antibody. Scalebar = 50 µm.

**Figure 2:**
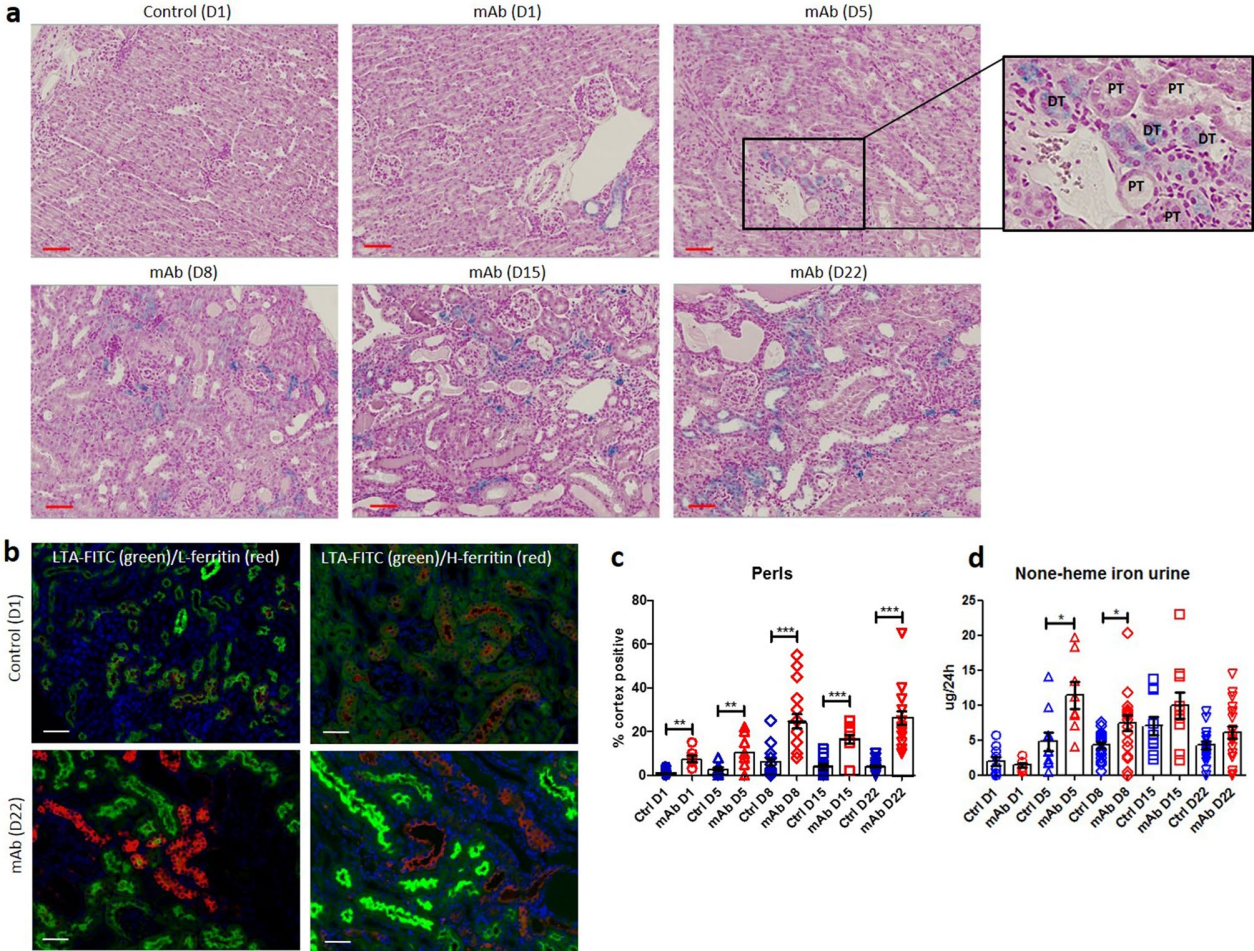
Kidney iron loading in mAb-injected mice. Representative images of kidney Perls staining demonstrating iron deposition (in blue) in mAb-injected mice that increases over time (**a**). Immunostaining of L-ferritin and H-ferritin in combination with proximal tubule marker LTA-FITC demonstrates distal tubule iron deposition (**b**). Quantification of kidney iron loading as a percentage of cortex tubules positive for iron deposition (**c**). Significantly increased urine non-heme iron levels were observed in mAb-injected mice compared to control at D5 and D8 (**d**). *p < 0.05, **p < 0.01, ***p < 0.001 compared to control using Mann–Whitney t-test; *D* day, *Ctrl* control, *mAb* monoclonal antibody, *PT* proximal tubule, *DT* distal tubule. Scalebar = 50 µm in A, 20 µm in (**b**).

**Figure 3:**
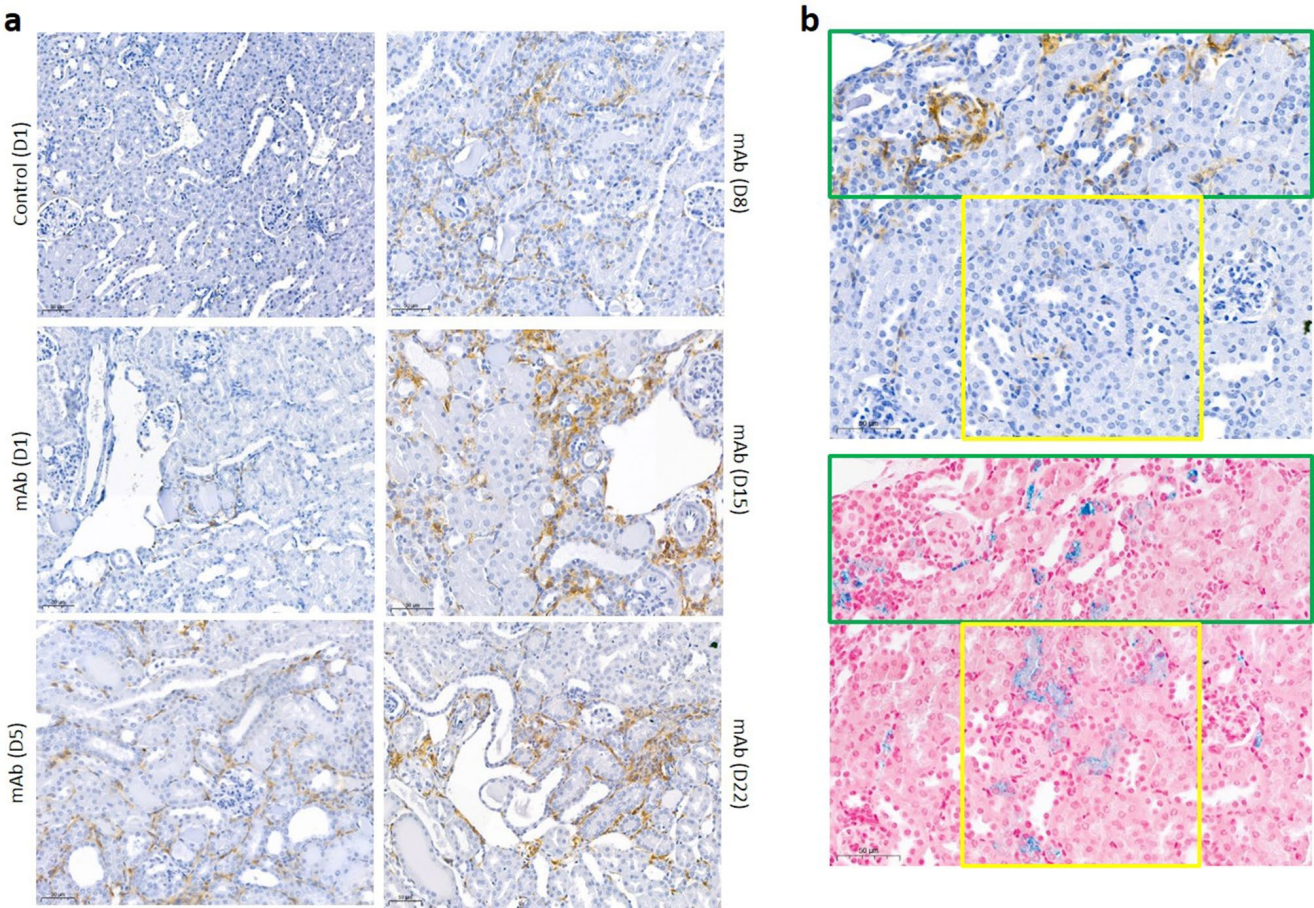
Macrophage influx and iron deposition in mAb-injected mice. Representative images of F4/80 immunostaining (brown) in mAb-injected mice that increased over time (**a**). Representative image of serial sections stained with F4/80 and Perls (**b**). The green square indicates an area of co-localization of macrophages and iron deposition; the yellow square demonstrates an area with only intratubular iron deposition, without macrophage influx. Scalebar = 50 µm.

Persistent proteinuria was confirmed in mAb-injected mice (Fig. 1a) by increased albuminuria at all time points (Fig. 1b). PAS staining showed progressive kidney injury in mAb-injected mice characterized by tubular dilatation and intratubular protein casts at D1 through D22 (Fig. 1c), and glomerular sclerosis from D8 onward. Kidney injury was further confirmed by significant increases in kidney mRNA expression level of *Interleukin-6* (*Il-6*) as a marker of kidney inflammation and *Heme oxygenase-1* (*Ho-1*), indicating oxidative stress (Fig. 1d). Urine markers for kidney injury (Fig. 1e) were also significantly elevated compared to control mice: 24p3 (NGAL/Lcn-2) and KIM-1. Whilst kidney iron deposition was absent or very limited in control mice, a time-dependent accumulation of kidney iron deposition could be observed in mAb-injected mice (Fig. 2a), which was predominantly located to cortical DT as indicated by the Perls staining. Also, L-ferritin and H-ferritin immunostaining in mAb-injected mice was mostly present in tubules that did not contain L-FITC, a marker for proximal tubules (Fig. 2b). The number of iron-positive tubules and urine non-heme iron content were significantly increased in mAb-injected mice compared to control (Fig. 2c,d). The progression of kidney injury was accompanied with an increased influx of macrophages as indicated by the F4/80 immunostaining (Fig. 3a). Nevertheless, staining of serial sections with F4/80 and Perls demonstrated that the iron deposition was not only localized with the extratubular macrophage influx but also present intratubular in areas without significant F4/80 staining (Fig. 3b). Combined, these results support increased exposure of the kidney to luminal iron as a result of proteinuria.

### Distal tubular iron loading in experimental FGSG is potentially mediated by 24p3R

**Figure 4:**
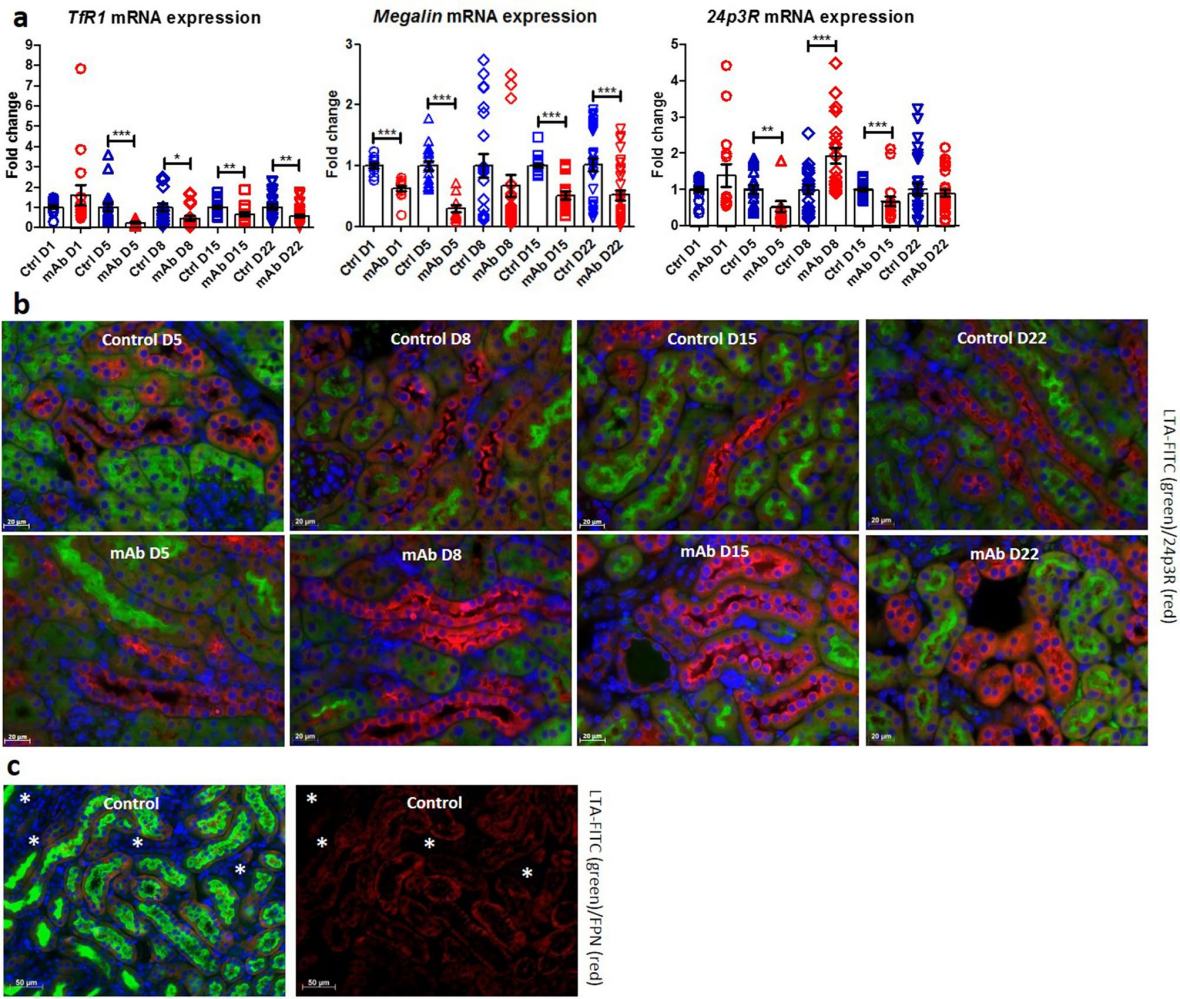
Kidney iron handling in mAb-injected mice. Reduced kidney mRNA expression of *transferrin receptor 1* (*TfR1*) and *megalin* in mAb-injected mice compared to control; *24p3R* was decreased at D5 and D15, but increased at D8 (**a**). Representative images of kidney 24p3R immunostaining (red) confirming distal tubular localization as its presence is not co-localized with proximal tubule marker LTA-FITC (green), showing increased intensity in mAb-injected mice compared to control (**b**). Representative image of immunostaining on ferroportin (FPN, in red) in control kidney, present at the basolateral membrane of LTA-FITC (green) positive proximal tubules (**c**). LTA-FITC negative distal tubules are indicated by the asterisks. *p < 0.05, **p < 0.01, ***p < 0.001 compared to control using Mann–Whitney t-test; *D* day, *Ctrl* control, *mAb* monoclonal antibody. Scalebar = 20 µm for (**b**) and 50 µm for (**c**).

Subsequently, we characterized the potential route of the DT iron loading observed in the mAb-injected mice. Kidney mRNA expression of *TfR1* was significantly decreased in mAb-injected mice compared to control from D5 onward (Fig. 4a), compatible with iron loading, since TfR1 expression is negatively regulated by intracellular iron stores via the iron response element-iron responsive protein (IRE-IRP) mechanism^34^. Also mRNA expression of *megalin* was significantly reduced in mAb-injected mice, whereas *24p3R* expression levels were slightly decreased at D5 and D15, but twofold increased at D8. Immunohistochemistry staining of 24p3R demonstrated increased abundance in mAb-injected mice compared to controls from D5 onward in the DT (shown for D5, D8, D15 and D22, Fig. 4b), suggesting potential for enhanced DT iron uptake. Moreover, FPN immunostaining demonstrated basolateral localization in PT, but absence from DT (Fig. 4c). Combined, these results suggest that the iron accumulation observed in the DT of mAb-injected mice could be mediated via 24p3R iron uptake and lack of cellular iron export mechanisms.

### Reduced kidney injury by iron-reducing interventions

**Figure 5:**
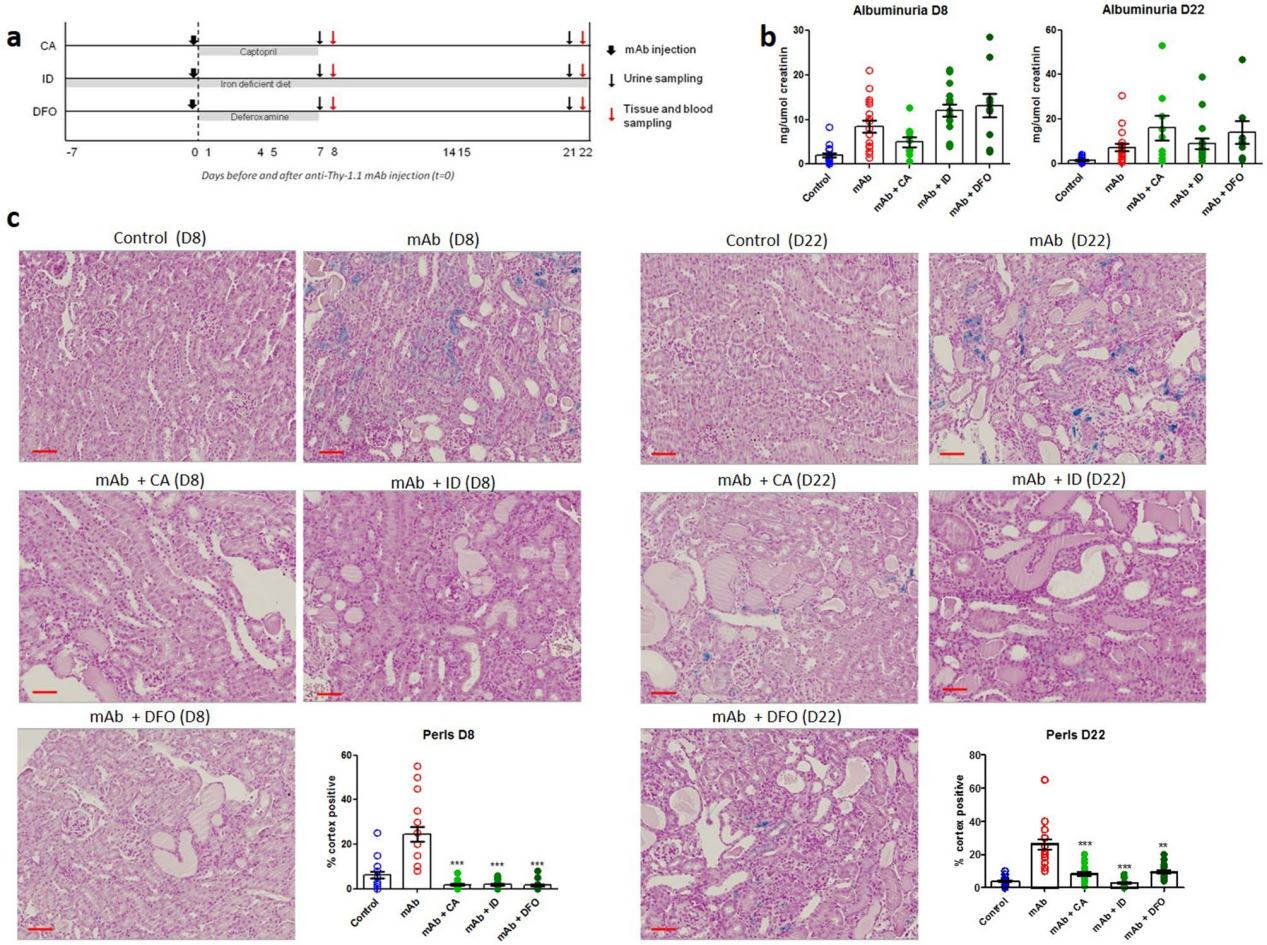
Iron-reducing interventions diminish kidney iron loading in mAb-injected mice. Schematic overview of experimental setup using CA, ID and DFO (**a**). Albuminuria was not significantly changed by CA, ID or DFO at D8 and D22 after mAb-injection compared to mice treated with mAb alone (**b**). Kidney iron deposition at D8 and D22 was significantly reduced in mAb-injected mice co-treated with CA, ID and DFO compared to mAb alone (**c**). ***p < 0.001 compared to mAb using one-way ANOVA Kruskal–Wallis test; *D* day, *mAb* monoclonal antibody, *CA* captopril, *ID* iron-deficient diet, *DFO* deferoxamine. Scalebar = 50 µm.

**Figure 6:**
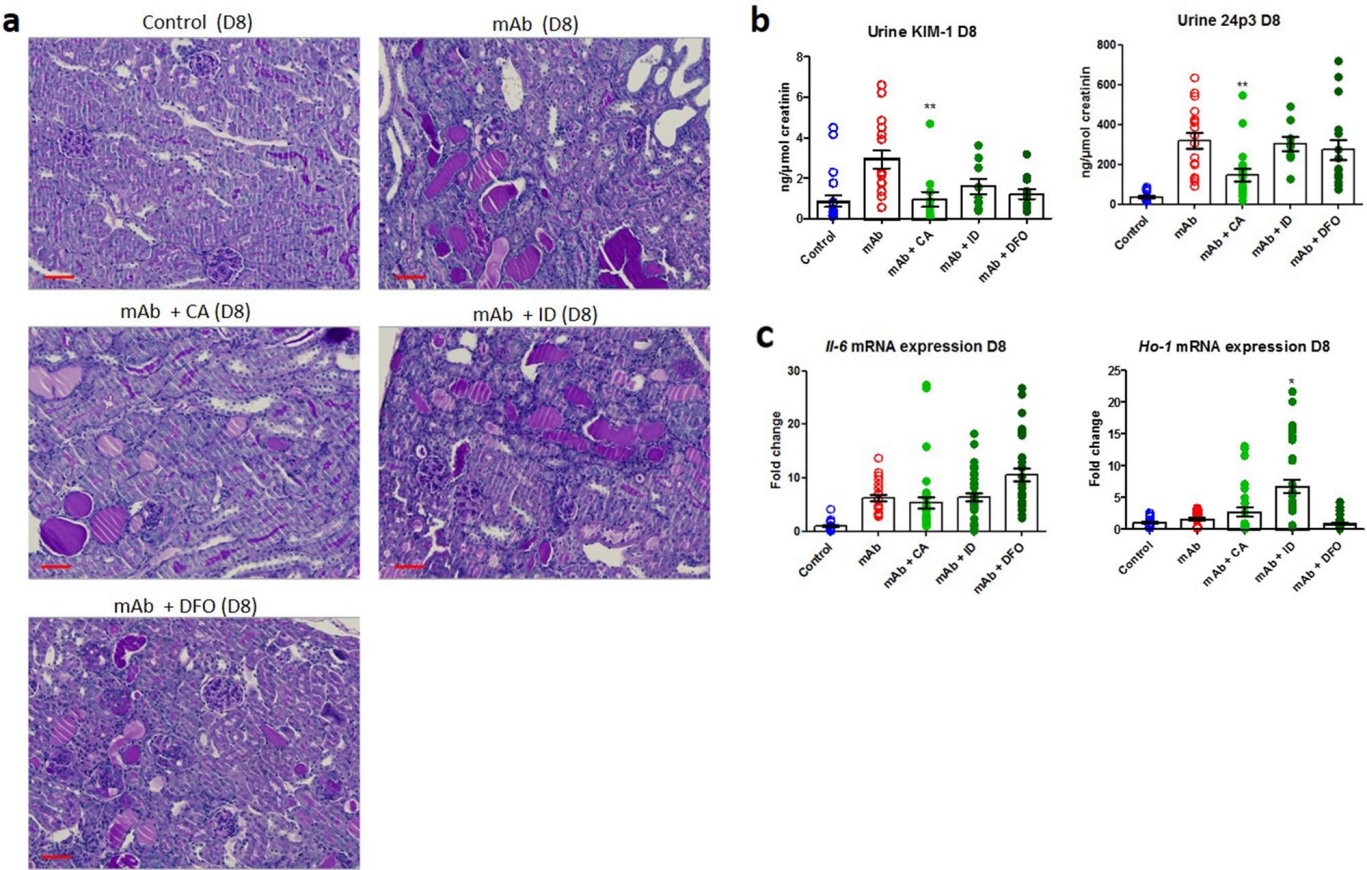
Reduced kidney injury by iron-reducing interventions at D8 in mAb-injected mice. Representative images of PAS staining in mAb-injected mice treated with CA, ID and DFO compared to mAb alone and control at D8 (**a**). Reduced urine KIM-1 concentration after CA and DFO, not significantly for ID, and reduced urine 24p3 concentration in mice treated with CA (**b**). Kidney mRNA expression of *Il-6* and *Ho-1* (**c**). No significant change in *Il-6* mRNA expression, reduced *Ho-1* mRNA expression after DFO treatment and increased *Ho-1* after ID. *p < 0.05, **p < 0.01, ***p < 0.001 compared to mAb using one-way ANOVA Kruskal Wallis test; *D* day, *mAb* monoclonal antibody, *CA* captopril, *ID* iron-deficient diet, *DFO* deferoxamine. Scalebar = 50 µm.

**Figure 7:**
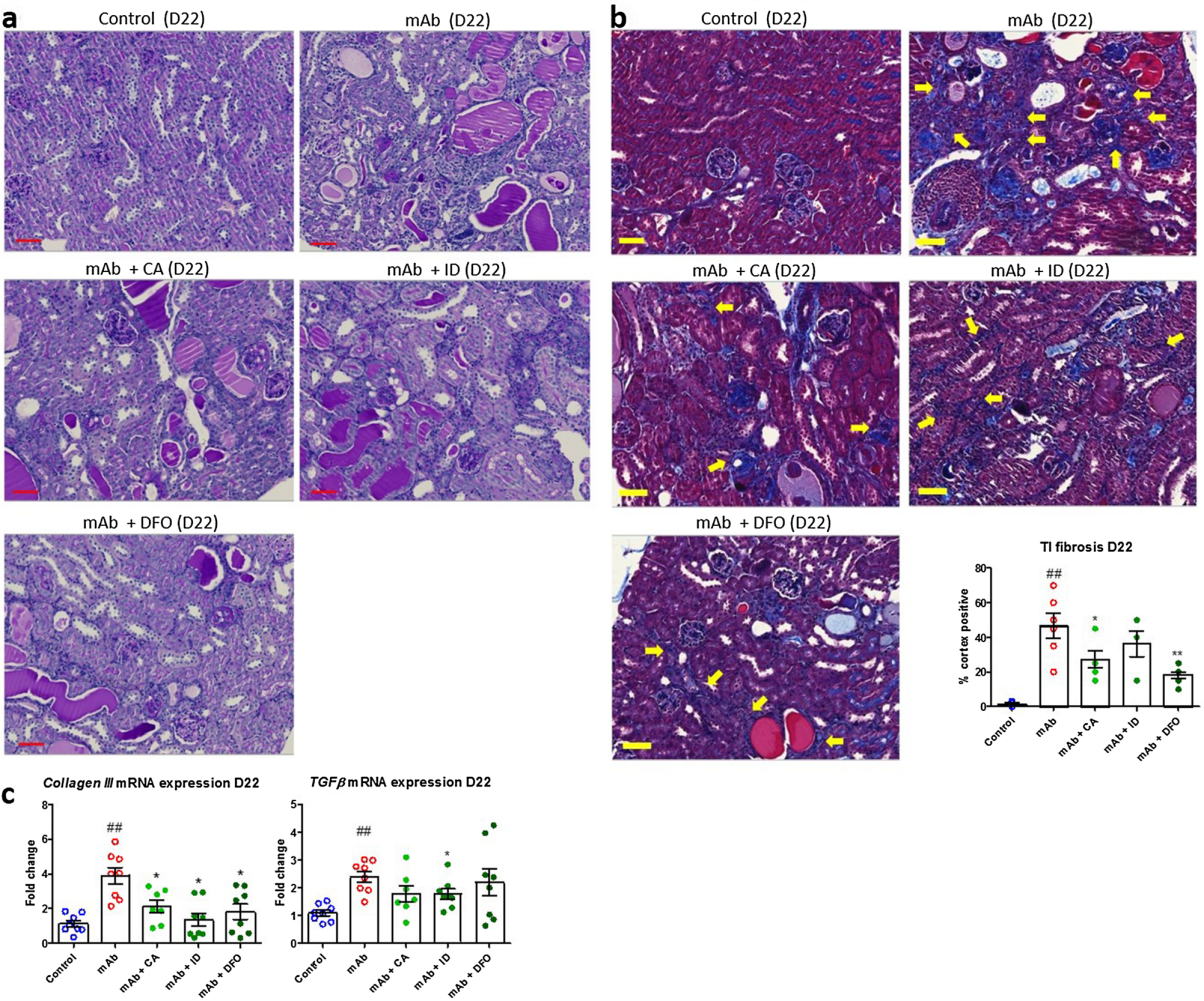
Reduced tubulointerstitial fibrosis by iron-reducing interventions at D22 in mAb-injected mice. Representative images of PAS staining in mAb-injected mice treated with CA, ID and DFO compared to mAb alone and control at D22 (**a**). Representative images of CAB staining in mAb-injected mice treated with CA, ID and DFO compared to mAb alone and control at D22 (**b**). Tubulointerstitial fibrosis (blue) is indicated by arrows and quantified in the graph (n = 4–8 per group). mRNA expression levels of *Collagen III* and *TGFβ* demonstrate a significant increase in mAb-injected mice compared to control, which is reduced by CA, ID and DFO (**c**). *p < 0.05, **p < 0.01 compared to mAb, ^###^p < 0.001 compared to control using one-way ANOVA Kruskal–Wallis test; *D* day, *mAb* monoclonal antibody, *CA* captopril, *ID* iron-deficient diet, *DFO* deferoxamine. Scalebar = 50 µm.

To investigate the contribution of kidney iron loading to the progression of TI injury, mAb-injected mice were subjected to three different iron-reducing strategies (Fig. 5a). ACE inhibitor CA was used to reduce proteinuria by reduction of blood pressure and, consequently, also glomerular filtration of iron; the ID diet was used to reduce total body iron levels, rendering less iron available for kidney deposition; and iron chelator DFO was administered to remove excess reactive iron from the kidney. The ID diet was provided throughout the entire duration of the experiment to ensure reduced body iron levels. Conversely, CA was given only for 7 days after mAb injection, since this regimen was proven successful to reduce proteinuria in Thy-1.1 mice previously^35^. DFO administration was also limited to 7 days after mAb injection to prevent potential nephrotoxicity. As expected, a reduction in albuminuria, although not significantly, could be observed at D8 by CA treatment, but remained unchanged after ID diet and DFO treatment (Fig. 5b). Kidney iron deposition was, however, significantly reduced by all interventions at both D8 and D22 after mAb-injection (Fig. 5c). Glomerulosclerosis and protein casts remained present in all treatment groups at D8 (Fig. 6a), since iron reduction was not expected to prevent or reduce glomerular damage. Nevertheless, diminished TI injury was suggested by reduced urinary KIM-1 concentration after CA treatment at D8 (Fig. 6b). ID diet and DFO show a trend towards reduced KIM-1 concentrations, but were not significant. Interestingly, differential effects were seen in the other injury parameters measured as well. Urinary 24p3 concentration was only reduced after CA treatment (Fig. 6b). Kidney *Il-6* mRNA expression remained elevated after all treatments at D8 (Fig. 6c). *Ho-1* mRNA expression was reduced after DFO treatment compared to mAb injection alone, whereas ID diet significantly increased kidney *Ho-1* mRNA expression (p < 0.001). At D22, glomerulosclerosis and protein casts were not reduced by any of the interventions (Fig. 7a), similar as for D8. Accordingly, at D22, urinary KIM-1 and 24p3 concentrations, as well as kidney mRNA expression levels of *Il-6* and *Ho-1* of mice treated with CA, ID diet or DFO did not differ from mice treated with mAb injection alone (data not shown). To investigate if the iron-reducing intervention had any beneficial effect on the progression of TI injury, kidney fibrosis was assessed by CAB staining and *Collagen III* and *TGFβ* mRNA expression levels. At D22, TI fibrosis was observed to a large extent in mice treated with mAb alone (Fig. 7b). Fibrosis percentage as measured by CAB staining was significantly reduced by CA and DFO. *Collagen III* mRNA expression was significantly increased in mAb-injected mice compared to control, and significantly reduced by all interventions. Lastly, *TGFβ* mRNA expression was significantly increased in mAb-injected mice compared to control, but only significantly reduced again by ID. Taken together, these results demonstrate that reducing kidney iron exposure strongly diminish kidney iron deposition. Despite variability in the different readouts, the data suggests that prevention of iron accumulation may reduce chronic kidney injury by means of reduced TI fibrosis.

## Discussion

Kidney iron loading has been demonstrated in proteinuric kidney diseases, but insights into the molecular mechanisms of iron accumulation and its contribution to progression of kidney injury remained elusive. Here, we discovered a mechanism of distal tubular iron loading as well as a complex role for iron in proteinuric kidney diseases.

In Thy-1.1 mice we predominantly observed iron accumulation in the DT. In contrast, we found iron deposition in both PT and DT in kidney biopsies of FSGS patients^36^. This discrepancy may be explained by the difference in disease progression at the time point of tissue collection between patients and Thy-1.1 mice. Disease progression in the Thy-1.1 mice is rapid compared to humans and the acute onset of proteinuria may lead to immediate high exposure of epithelial cells to TBI filtered by the glomerulus. TBI is likely reabsorbed by the PT due to its large iron reabsorption capacity by TfR1 and megalin. PT iron uptake in mAb-injected mice was confirmed by reduced *TfR1* expression, in line with increased intracellular iron levels by means of IRE/IRP regulation^34^. The relative minor PT iron staining may be explained by the presence of FPN. Nevertheless, the increased urine concentration of PT injury marker KIM-1 and the reduced expression of *megalin* in the mAb-injected Thy-1.1 mice suggests PT injury, especially in the early phase of disease progression^37^. Consequently, more iron is available at the site of the DT and might be reabsorbed by 24p3R. Moreover, the absence of FPN in the DT promotes intracellular iron accumulation. Gradual disease progression in humans may result in lower but steady long-term TBI exposure of the PT, resulting in detectable iron accumulation. In our studies, we did not investigate the potential role of divalent metal transporters in kidney iron transport in our experimental FSGS model. However, with a presumed role for 24p3R in DT iron uptake, an additional role for any divalent iron transporter, such as DMT-1, ZIP14 or ZIP8, might be expected^38^. To evaluate this interesting proposed route of DT iron loading, future studies focused on functional evaluation of iron transport are required.

Increasing kidney iron accumulation coincided with the progression of histological kidney injury and elevation of urinary KIM-1 and 24p3 levels, and kidney *Il-6* and *Ho-1* mRNA expression. Ca, ID diet and DFO all, albeit to various degrees, lowered kidney iron exposure and also reduced kidney injury at D8, as indicated by diminished urinary KIM-1 levels, and at D22, indicated by reduced TI fibrosis staining, *Collagen III* and *TGFβ* mRNA expression. The reduction in kidney iron loading by CA confirms that kidney iron deposition during FSGS originates from proteinuria. Remarkably, the three iron-reducing interventions showed a differential response in injury parameters. Urine 24p3 concentration was only reduced by CA treatment, suggesting that the reduction of proteinuria altogether by CA had a greater beneficial effect on urine 24p3 than the reduction in kidney iron exposure specifically. Nevertheless, our results suggest that interventions specifically aimed at reducing kidney iron loading can be further explored as an alternative strategy for patients with glomerular injury that do not benefit sufficiently from ACE inhibition.

Interestingly, there was no beneficial effect of iron reduction on kidney inflammation by any of the interventions, which may be in line with previous studies of Vaugier et al*.* who demonstrated that sufficient systemic iron levels are necessary to protect against ischemia–reperfusion injury-associated sterile inflammation^39^. Moreover, the increased expression of kidney *Ho-1* after ID diet suggests that too much reduction in kidney iron content and/or systemic iron levels may be detrimental. Although HO-1 has been recognized as a protective mediator in kidney injury, it can also be a marker of tubular stress^40^. Since we observed a marked increase in *Ho-1* mRNA expression as a marker of kidney injury in the mAb-injected mice, we interpreted the further increase in *Ho-1* mRNA expression after ID diet as an indicator of tubular stress. Indeed, the PT epithelial cells have a high expression of HO-1, high energy demand and are abundant in mitochondria, which require iron for energy metabolism^41,42^. It was demonstrated in cultured PT epithelial cells that the nephrotoxicity of iron chelator deferasirox may be due to cellular iron depletion, since loading of deferasirox with iron before cell incubation abolished its toxic potential^43^. In our study, we administered DFO for only 7 days after mAb injection to minimize DFO-mediated toxicity. Our results demonstrate that removal of excess iron by chelation is preferred over reducing total body iron levels by ID diet as indicated by the reduced *Ho-1* mRNA expression after DFO treatment compared to mAb injection alone. Despite the promising protective effects of 7 day DFO treatment on short term injury and TI fibrosis, prolonged DFO treatment is not feasible due to its nephrotoxic potential. Combined, these results indicate that there is a delicate iron balance in the kidney that needs to be maintained for adequate functioning and protection, which may differ between the PT and DT. This warrants an approach to remove or prevent excessive iron accumulation, without affecting the minimal iron requirement or systemic iron balance. It remains unclear if iron-mediated injuries in the PT and DT both contribute to TI fibrosis. To evaluate this, future studies particularly targeting the proximal or distal tubule iron uptake mechanisms, for example using a genetic (inducible) 24p3R knockout model, combined with a model that allows for long term follow up of iron loading during disease progression, for instance by inducing adriamycin-mediated FSGS^44^, are needed. Another avenue for targeted intervention may be found by exploring the detrimental effects of kidney iron loading in molecular detail. In recent years, it has become evident that iron plays an important role in a form of regulated necrosis, called ferroptosis^45^. Interestingly, kidney tubule epithelial cells are sensitive to ferroptotic cell death, which was demonstrated to be involved PT cells in various preclinical models of acute kidney injury^46^. Given the combined evidence of kidney iron accumulation in CKD and the association between ferroptosis and fibrosis^47^, it might be worthwhile to investigate the role of ferroptosis in CKD and DT injury.

Taken together, the results of our study demonstrate that kidney iron loading occurs in concordance with progressive TI injury in experimental FSGS and that reduction of kidney iron loading can halt TI fibrosis. The results also implicate that a targeted approach that reduces kidney iron loading, but maintains minimally required cellular iron levels, is warranted to maintain an optimal kidney iron balance.

## Methods

### Animal studies

All experiments were approved by the local Animal Welfare Ethics Committee of the Radboudumc (Nijmegen, the Netherlands; DEC 2015-0084) in accordance with the guidelines of the Principles of Laboratory Animal Care (NIH publication 86–23, revised 1985), and reported in as described by the ARRIVE guidelines (https://arriveguidelines.org/). Thy-1.1 mice were housed under controlled conditions with standard chow and water ad libitum, unless stated otherwise. At 6 weeks of age male and female Thy-1.1 mice received a tail vein injection of 1 mg anti-Thy-1.1 monoclonal antibody (mAb; 19XE5) in 0.1 mL 0.9% saline or vehicle as control^33^. Mice were sacrificed on day 1 (D1), D5, D8, D15 or D22 after mAb injection and plasma was collected (Fig. 1A). Prior to sacrifice, a 24 h urine sample was collected by a metabolic cage. Tissues were collected in liquid nitrogen and stored at − 80 °C or in 4% formalin O/N before embedding in paraffin.

Experimental groups included n = 10 per group for D1, D5 and D15, and n = 20 per group for D8 and D22. Data collected at D8 and D22 were used as controls for subsequent interventions using ACE inhibition, iron-deficient diet and iron chelation as described below.

Iron-deficient (ID) diet (2–6 ppm iron) was given to the mice from one week prior to mAb injection until sacrifice at D8 or D22 (n = 18 per group). Captopril (CA) was administered via the drinking water (400 mg/L) ad libitum for 7 days after mAb injection (n = 18 per group). Iron chelation by deferoxamine (DFO) was performed by daily i.p. injections of 100 mg/kg/day for 7 days after mAb injection (n = 18 per group).

### Urine albumin measurement

Urine albumin was measured by radial immunodiffusion using a goat antiserum against mouse albumin^33^.

### Creatinin determination

Urinary creatinin concentration was determined using the assay kit based on the Jaffé method from Labor & Technik (LT-SYS 0251).

### Histology, immunohistochemistry and immunofluorescence

Periodic Acid Schiff (PAS) staining, Perls Prussian blue staining and Chromotrope Aniline Blue (CAB) staining were performed by routine assays at the pathology laboratory of the Radboudumc (Nijmegen, the Netherlands). Images were taken using the VisionTek^®^ Digital Microscope (Sakura).

For immunohistochemistry, kidney sections were deparaffinized and an antigen retrieval step was performed using citrate buffer pH6, followed by washing with 0.1% PBS-Tween. Sections were then incubated with 1% BSA in PBS-Tween for 1 h, followed by the first primary antibody O/N at 4 °C: rabbit polyclonal anti-24p3R antibody (CT-terminal, #1086^24^) at 1:500 dilution, rabbit polyclonal anti-FPN (ab85370, Abcam) at 1:500 dilution, rabbit polyclonal anti-L-ferritin (69090, Abcam) at 1:1000 dilution, or rabbit polyclonal anti-H-ferritin (65080, Abcam) at 1:200 dilution. The secondary goat-anti-rabbit Alexa Fluor 568 antibody (Invitrogen) was added at a 1:200 dilution for 30 min at RT together with 1:100 FITC-labeled Lotus Tetraglonolobus Agglutinin (LTA-FITC; Vector Laboratories). For nuclear staining DAPI (1:1000) was added for 5 min. Sections were fixed with fluorescent mounting medium (DAKO). Fluorescent staining was visualized using an Apotome.2 FL microscope (Zeiss). For F4/80 staining, sections were incubated with the primary antibody rabbit monoclonal F4/80 antibody (70076, CellSignal) at 1:250 dilution. The F4/80 signal was detected using a biotinyl∆ated goat-anti-rabbit IgG antibody (Vector Laboratories, BA-1000) and the VECTASTAIN^®^ Elite^®^ ABC-HRP kit (Vector Laboratories, PK-6100). The 3,3′-diaminobenzidine (DAB) horseradish peroxidase was used as substrate.

### RNA isolation and quantitative PCR

RNA was isolated with TRIzol (Life Technologies), according to manufacturer’s instructions. Quantitative PCR was performed with SYBR Green mastermix (2x; Applied Biosystems) and primers are listed in Table 1. Fold change values compared to control or baseline were calculated with the 2^−∆∆Ct^ formula.

**Table 1:** Primers.

Gene	Forward primer (5ʹ–3ʹ)	Reversed primer (5ʹ–3ʹ)
*β-actin* (housekeeping gene)	GCTATGCTCTCCCTCACGCCA	CTCTTTGATGTCACGCACGAT
*24p3R*	GATAGACAGGAAGGCAAGGC	GACGGAGTGAACAGAAAGCA
*IL-6*	GAGGATACCACTCCCAACAGACC	AAGTGCATCATCGTTGTTCATACA
*Ho-1*	CCTCACTGGCAGGAAATCAT	CCAGAGTGTTCATTCGAGCA
*Megalin*	CCTCCTTCACCTGCGACAAT	TCCCCGAAGCTTGACAGTTC
*TfR1*	CGCTTTGGGTGCTGGTG	GGGCAAGTTTCAACAGAAGACC
*TGFβ*	GCAGTGGCTGAACCAAGGA	AAGAGCAGTGAGCGCTGAATC
*Collagen III*	GCAATATGCCCACAGCCTT	ATTTCTCCCAGGAATGCCA

### ELISA

Concentrations of 24p3 and KIM-1 were determined in urine using the DuoSet ELISA development kits from R&D systems (DY1857 and DY1817) according to manufacturer’s protocol.

### Non-heme iron assay

Total non-heme iron levels were measured in urine using the bathophenanthroline assay (adapted from Torrance and Bothwell)^48^.

### Statistical analysis

Data were statistically analyzed using GraphPad Prism 5.03 software (GraphPad Software, La Jolla, CA) and presented as means ± SEM. Results were analyzed for statistically significant differences using the t-test or one-way ANOVA with post hoc analysis wherever appropriate as indicated in the figure legends. A p-value of < 0.05 was considered statistically significant.

## Data Availability

The datasets generated during and/or analysed during the current study are available from the corresponding author on reasonable request.
